# High Phosphate Load Induces De Novo Formation of Tertiary Lymphoid Structures in the Kidney

**DOI:** 10.1096/fj.202500968R

**Published:** 2025-12-12

**Authors:** Nina Weingärtner, Beatrice Richter, Franziska Walles, Tamar Kapanadze, Jessica Schmitz, Jan H. Bräsen, Florian P. Limbourg, Dieter Haffner, Maren Leifheit‐Nestler

**Affiliations:** ^1^ Department of Pediatric Kidney, Liver, Metabolic and Neurological Diseases, Pediatric Research Center Hannover Medical School Hannover Germany; ^2^ Department of Nephrology and Hypertension Hannover Medical School Hannover Germany; ^3^ Vascular Medicine Research Hannover Medical School Hannover Germany; ^4^ Institute of Pathology, Nephropathology Unit Hannover Medical School Hannover Germany

**Keywords:** fibroblast growth factor 23 (FGF23), fibrosis, inflammation, kidney injury, phosphate, tertiary lymphoid structures

## Abstract

Tertiary lymphoid structures (TLS) are associated with inflammatory kidney diseases, but their pathogenesis is unclear. A chronic high phosphate diet (HPD) in mice increases serum levels of phosphate and fibroblast growth factor 23 (FGF23) which is associated with progressive tubule damage and fibrosis. We hypothesized that chronic HPD induces TLS in the kidneys and thereby promotes progressive kidney injury. After only 2 months, mice on HPD showed increased tubule damage accompanied by the formation of perivascular immune cell clusters of B and T cells in the corticomedullary zone and in the cortex. In addition, lymphatic vessels were observed, accompanied by increased expression of venous markers and cell adhesion molecules. Further analyses showed a time‐dependent induction of the immunofibroblast‐derived chemokines and lymphotoxins, which are important for the differentiation of immunofibroblasts into follicular dendritic cells (FDC) and fibroblastic reticular cells (FRC). Already after 4 months of HPD, proliferating B‐cell clusters with FDCs, T‐cell clusters with FRCs, podoplanin^+^ cellular networks, high endothelial venules, plasma cells and increased IgD synthesis indicated fully mature TLSs, while tubular damage and fibrosis continued to increase up to 6 months of feeding HPD. Genetically modified mice overexpressing FGF23 developed tubular damage, fibrosis, and fully mature TLS only in the presence of HPD‐induced hyperphosphatemia. Likewise, hypophosphatemic *Hyp* mice showed no signs of tubular damage or TLS despite increased FGF23 levels. Our data suggest that high phosphate directly causes chronic inflammation in the kidney leading to the development of fully mature TLS associated with progressive tubular injury.

## Graphical abstract

Graphical illustration of renal alterations under varying phosphate load: High phosphate load induces fibrosis, tubular injury, impaired renal function, and de novo formation of perivascular tertiary lymphoid structures. Under normal phosphate load, fibrosis, functional decline, and tubular injury are absent, while T‐ and B‐ cell aggregates are present without TLS formation. Low phosphate load is associated with preserved renal integrity and absence of TLS development.

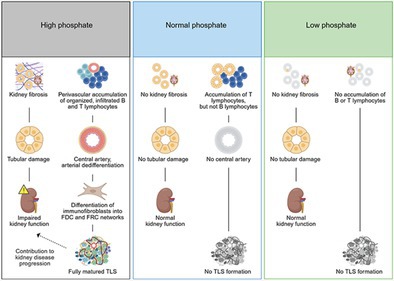

## 1 Introduction

Tertiary lymphoid structures (TLS) are immune cell aggregates developing in nonlymphoid tissues by various stimuli including chronic inflammation initiating adaptive immune responses [1, 2, 3]. In humans, TLS have been observed in various pathologies and organs [4, 5, 6, 7, 8, 9]. Renal TLS formation was mainly described in patients with inflammatory kidney diseases [10, 11, 12, 13, 14, 15] and noted in experimental models of ischemic reperfusion injury, unilateral ureteral obstruction, folic acid‐induced nephropathy, and in aging kidneys [11, 16]. Mature TLS are characterized by well‐organized T and B cell compartments, a follicular dendritic cell (FDC) network in B cell areas, a network of fibroblastic reticular cell (FRC) in T cell areas, the presence of plasma cells, and evidence of B cell class switching and germinal center‐like structure formation [17, 18, 19]. The pathogenesis of TLS generation is poorly understood and it is not clear whether renal TLS develop independent of organ damage or as a response to cellular injury further promoting kidney disease progression [11, 12, 20, 21]. A recent preclinical study demonstrated that the loss of Notch signaling in vascular endothelial cells triggers the formation of periarterial TLS in mouse kidneys, causing arterial vessels to adopt a high endothelial venules (HEV) phenotype and enhance lymphocyte recruitment, which are the early features of TLS formation [22]. Other studies indicate that fibroblastic priming and transdifferentiation of immunofibroblasts into FRC and FDC networks are pivotal drivers in the formation and development of TLS [23]. Findings from micro‐dissected TLS of lupus nephritis patient kidney biopsies detected clonal B cells and a germinal center‐like structure response with autoantibody generation, which could potentially contribute to kidney disease progression by binding proximal tubular cells and thereby stimulating cytokine production [12, 24]. Moreover, these patients showed follicular T cell‐like CD4^+^ cells in the kidney, which were associated with a lower estimated glomerular filtration rate (eGFR), underlining the hypothesis of a more pathologic contribution of TLS in this disease.

Chronic kidney disease (CKD) results in hyperphosphatemia, enhanced tubular phosphate load and increased synthesis of fibroblast growth factor 23 (FGF23), which is discussed to contribute to inflammation and disease progression [25, 26, 27, 28, 29]. We have recently shown that chronic high phosphate load in mice leads to hyperphosphatemia with increased FGF23 levels and progressive tubular injury, tubulointerstitial fibrosis, and kidney inflammation [30]. Here, we use wildtype and genetically modified mice to evaluate the causal contribution of high phosphate or enhanced FGF23 levels on tubular injury, fibrosis and TLS formation and to analyze whether TLS development is directly associated with kidney damage. Our results provide important evidence that high phosphate, rather than high FGF23, promotes an inflammatory environment that leads to TLS formation and progressive kidney injury, further emphasizing the pathophysiological role of high phosphate in the pathogenesis of kidney injury and the progression of CKD.

## 2 Methods

### 2.1 Animal Experiments

The high phosphate‐induced mouse model of kidney injury, tubulointerstitial fibrosis and inflammation was conducted in accordance with previous studies [30]. Briefly, *n* = 100 8‐week‐old male C57BL/6NCtrl mice (Charles River) were block randomly divided into two groups with each *n* = 5 animals per cage, and each group was administered either a 2% HPD (#C1049) or a 0.8% Ctrl (#C1000, both Altromin), respectively. After 1, 2, 3, 4, and 6 months each *n* = 10 animals per group were used for analyses. To specifically enhance circulating iFgf23 levels, 8‐week‐old male C57BL/6NCtrl mice (Charles River) received an adeno‐associated virus serotype 9 in which the full‐length cDNA of murine Fgf23 (AAV‐Fgf23) was subcloned as described previously [31]. Briefly, *n* = 24 mice were block randomly divided into three groups with each *n* = 4 animals per cage. Two groups were injected subcutaneously with 5 × 10^11^ vg AAV‐Fgf23 and one group with AAV‐vehicle as control (AAV‐Ctrl) [32]. Two weeks postinjection each *n* = 8 mice with AAV‐Ctrl and AAV‐Fgf23 received the Ctrl diet and *n* = 8 AAV‐Fgf23 mice received the HPD for 6 months. As an in vivo model for hypophosphatemia, each *n* = 8 8‐week‐old male B6.Cg‐*Phex* ^*Hyp*^/J mice (*Hyp*, strain # 000528, The Jackson Laboratory, RRID:IMSR_JAX:000528) and WT littermates both on normal rodent chow were used. No animals were excluded during the experiments. At the end of each experiment, spot urine was sampled, blood was collected by cardiac puncture under isoflurane anesthesia and serum and plasma were prepared, and kidneys, heart and liver were isolated. The poles of the kidneys were cut off and the middle disk of the left kidney was snap‐frozen in liquid nitrogen and stored at −80°C for further molecular and biochemical analyses. For histology, the middle disk of the right kidney, the mid cross‐section of the heart and liver tissue were fixated in 4% RotiHistofix (Carl Roth) for 24 h and embedded in paraffin. For flow cytometry analysis, half of the left kidney was used. All postmortem experiments were conducted in a blinded manner until the quantification and statistical analysis.

### 2.2 Study Approval

All animal experiments were approved by the Lower Saxony State Office for Consumer Protection and Food Safety (LAVES; IACUCs 17/2717, 15/1912, 11/0486) and performed according to the national animal protection guidelines from Directive 2010/63/EU of the European Parliament on the protection of animals used for scientific purposes. All animals were maintained in temperature‐controlled IVC cages with a 14/10‐h light/dark cycle and allowed ad libitum access to food and water.

### 2.3 Blood and Urine Analyses

Phosphate and creatinine in serum and urine as well as albumin in urine were measured by an enzymatic colorimetric method using the Cobas c111 automatic analyzer and Roche reagents (Roche). Plasma iFgf23 concentrations were determined via mouse/rat iFGF23 ELISA (Quidel) on a TECAN Infinite 200 microplate reader and quantified using Magellan software (Tecan).

### 2.4 Histological Staining

Two μm thick formalin‐fixed paraffin‐embedded kidney, liver and heart sections were deparaffinized, rehydrated through a graded ethanol series, and hematoxylin and eosin (HE), Periodic acid–Schiff (PAS), and picrosirius red staining were performed according to standard protocols. Antigen retrieval was done in 0.01 M citrate buffer, pH 6.0 for 20 min at 98°C. For IF staining, blocking with 5% BSA in 1× PBS was done. Incubation with primary antibodies (Table S1) and incubation with secondary antibodies (Table S2) were performed for 1 h at room temperature each, followed by nuclear counterstaining using DAPI containing mounting medium (scr‐038448, Dianova). For IHC staining, deparaffinization, antigen retrieval, and blocking of endogenous peroxidase activity were performed according to standard protocols, followed by incubation of the slides with the indicated antibodies (Tables S1 and S2). Subsequently, slices were treated with EnVision FLEX diaminobenzidine substrate + Substrate Chromogen System (GV825, Dako Omnis), followed by counterstaining with Mayer's hematoxylin (Merck) and mounting with Roti‐Mounting medium (HP68.1, Carl Roth). Images were taken with either 5× or 20× air objective or 40× oil objective using the Axio Observer Z1 microscope (Carl Zeiss, RRID:SCR_021351).

### 2.5 Quantification of Histological Stainings

TLS size was determined on HE‐stained kidney cross‐sections from three animals per group. TLS as well as total kidney areas were measured using ImageJ software (Fiji; RRID:SCR_003070) and TLS size was calculated as the proportion of the total kidney cross‐section area. F4/80^+^ macrophages were assessed by analyzing three representative images per TLS region per animal. The F4/80^+^ area was measured using ImageJ and expressed as a percentage of high‐power field (hpf).

For histological quantification of cells positive for CD45R, CD3, CD138, IgG, IgD, peripheral node addressin (PNAd), and lymphatic vessel endothelial receptor 1 (LYVE‐1), three representative images per TLS region per animal were acquired. Both single‐positive and double‐positive cells were manually counted using ImageJ. Double‐positive cells were expressed as a percentage of either CD45R^+^ or CD138^+^ cells, depending on the staining.

Fibrosis within TLS region was quantified on picrosirius red stained sections. For each animal, three representative hpf images were analyzed, and fibrotic areas were measured using ImageJ and expressed as a percentage of hpf.

For the quantification of tubular injury PAS and HE stained cross‐sections were blinded and evaluated by a nephropathologist, utilizing a scoring system that ranged from absence of damage (score 0) to pronounced damage (score 10). To quantify tubulointerstitial fibrosis, five images from randomly chosen cortical regions of picrosirius red stained kidney cross‐sections per mouse were captured and the fibrotic area per hpf was determined in percent using ImageJ.

### 2.6 RNA Isolation and Quantitative Real Time PCR (qRT‐PCR)

30 mg snap‐frozen kidney or liver tissue, respectively, was used for total RNA isolation utilizing the RNeasy Mini Kit according to manufacturer instructions, and 500 ng total RNA was transcribed into cDNA via the QuantiTect Reverse Transcription Kit (both Qiagen). qRT‐PCR was performed in triplicates using PowerTrack SYBR Green Master Mix (A46012, Applied Biosystems) on a QuantStudio 6 Pro system (Applied Biosystems, RRID:SCR_020239). Primer sequences are shown in Table S3. *Gapdh* was used as a housekeeping gene and relative mRNA expression was calculated according to the 2^−ΔΔCt^ method [33].

### 2.7 Protein Isolation and Mouse Inflammation Antibody Array

For mouse inflammation antibody array (ab133999, Abcam), 30 mg snap‐frozen kidney tissue was homogenized in 2× Cell Lysis Buffer (ab133999, Abcam) using the TissueLyser LT (Qiagen). Protein concentration was determined using the BCA assay (Thermo Fisher Scientific) according to the manufacturer's protocol. 250 μg of total protein was used for each membrane to detect inflammatory proteins simultaneously according to the manufacturer's protocol. Chemiluminescence signal detection was done with Pierce ECL Western Blotting Substrate (Thermo Fisher Scientific) on an Odyssey Imager (LI‐COR Biosciences, RRID:SCR_023227) and spot intensity was quantified with Image Studio 5.2 software (LI‐COR Biosciences). Quantification of signal was done according to the manufacturer's instructions.

### 2.8 Flow Cytometry

Single‐cell suspensions were prepared from the kidneys, using collagenase type 2 digestion, as described [30]. The cells were counted by Countessa II automatic counter (ThermoFisher Scientific, Waltham, MA, USA). Trypan blue (Roth, Karlsruhe, Germany) was used to define dead cells. The cell pellets were resuspended in the staining buffer: DPBS (Sigma Aldrich) with 2% fetal calf serum (Biochrom, Berlin, Germany), 2 mM Na_2_EDTA (Roth) and 0.02% NaN_3_ (AppliChem, Darmstadt, Germany). 10^6^ cells were taken for staining. TrueStain FcX anti‐CD32/16 (Biolegend, SanDiego, CA, USA) was used to block Fc receptors. Antibodies and streptavidin compounds for flow cytometry are listed in Table S4. The dead cells were excluded using propidium iodide (Fluka, Seelze, Germany) staining. The flow cytometry analysis was performed using LSR II flow cytometer and FlowJo software (both BD Biosciences, Heidelberg, Germany, RRID:SCR_008520). The relative frequencies of each subpopulation were defined within the live cell gate and shown in the graphs as mean ± SEM.

### 2.9 Statistical Analysis

Data are presented as mean ± SD. To identify outliers, the ROUT method with a Q of 1% was performed and cleaned data were tested for Gaussian distribution using Shapiro–Wilk (*n* < 8) or D'Agostino and Pearson (*n* > 8) normality test. Two‐tailed *t*‐tests of two unpaired groups were applied for normally distributed data, whereas non‐Gaussian distributed data were calculated using the Mann–Whitney *U* test. To analyze three groups, one‐way ANOVA (analysis of variance) or the Kruskal–Wallis test, followed by Dunn's or Sidak's *post hoc* tests was performed, respectively. For the time‐dependent alterations in array analysis ordinary ANOVA was used to identify significant differences among means. For all analyses, significance was presumed at *p* < 0.05. GraphPad Prism software version 10 (RRID:SCR_002798) was used for all statistical analyses.

## 3 Results

### 3.1 High Phosphate Load Induces the Perivascular Formation of Organized B and T Cell Infiltrates in the Kidney

**FIGURE 1:**
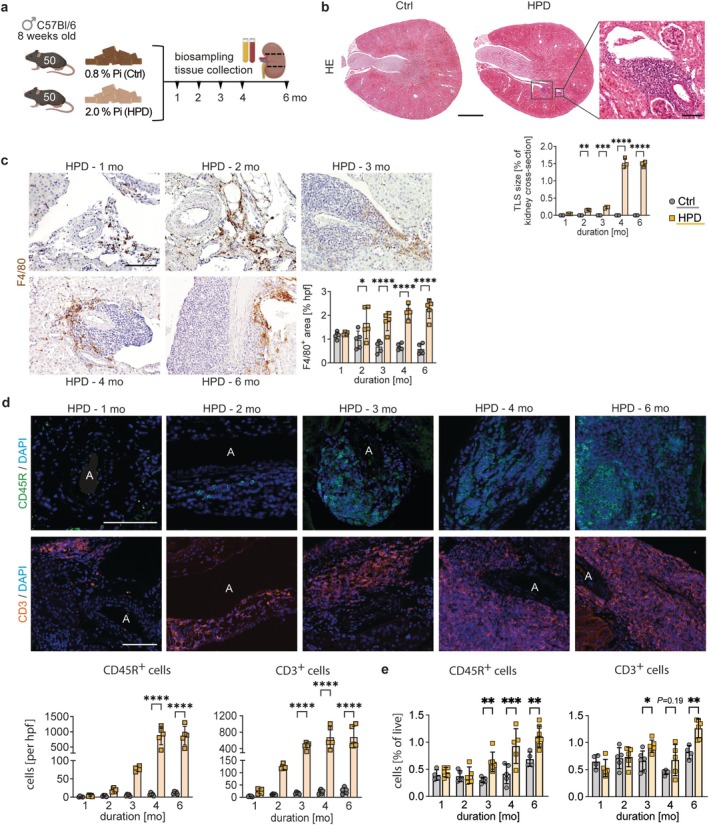
Chronic high phosphate load induces kidney‐specific tertiary lymphoid structures (TLS). (a) Schematic representation of the study design including dietary intervention and the specific time points for biosampling and tissue collection. (b) Representative hematoxylin and eosin (HE) stained section of an entire kidney cross‐section from a mouse fed with a control diet (Ctrl) or a high phosphate diet (HPD) for 6 months showing the presence of tertiary lymphoid structures (TLS) formed around a central artery in the corticomedullary zone only in HPD fed mice. Scale bars: 1 mm and 100 μm, respectively. TLS size was quantified as the percentage of the kidney cross‐sectional area in both groups. (c) Representative immunohistochemical staining for F4/80^+^ macrophages (brown) in kidney sections from mice that have received HPD for 1–6 months. Scale bar: 50 μm. F4/80^+^ area (% per hpf) was quantified in HPD and Ctrl group. (d) Time course of immunofluorescence staining of CD45R^+^ B cells (green) and CD3^+^ T cells (orange), with DAPI counterstaining (blue). Scale bars: 100 μm and 50 μm, respectively. Quantification of CD3^+^ T cells and CD45R^+^ B cells per hpf was performed. (e) Quantitative flow cytometry analysis of B cells and CD3^+^ T cells in whole kidney tissue of Ctrl and HPD mice. (a–e) Data are presented as mean ± SD. One‐way analysis of variance (ANOVA) with Sidak's multiple comparisons test, *p < 0.05; **p < 0.01; ***p < 0.001; ****p < 0.0001. A, artery.

Male C57BL/6N mice received either a 2% HPD or 0.8% phosphate control diet (Ctrl) for 1–6 months, and blood, urine and kidney tissue were collected every 4 weeks (Figure 1a). As previously published [30], HPD resulted in increased serum phosphate levels, enhanced urinary phosphate excretion, elevated plasma intact Fgf23 (iFGF23) concentrations, and a progressive impairment of kidney function with tubular injury and interstitial fibrosis already starting in mice after 1 month of HPD compared to respective Ctrl groups (Table 1). Histopathological analysis of HE‐stained kidneys after 6 months of HPD feeding showed large perivascular infiltrating mononuclear cell clusters in the corticomedullary zone in the area of the renal pelvis (Figure 1b). Intriguingly, this was not observed in the kidneys of Ctrl mice. The quantification of TLS size revealed that a significant difference in TLS size between HPD and Ctrl groups was already apparent after 2 months (Figure 1b). This was even more pronounced after 4 and 6 months of HPD feeding compared to Ctrl. Quantification of the number of TLS revealed a mean of 1.5 ± 0.5 TLS per kidney cross‐section of mice on HPD versus no TLS in the Ctrl group (*p* = 0.002). Moreover, these inflammatory cell clusters were kidney specific and were neither found in HE‐stained liver nor heart tissue from HPD mice (Figure S1a,b). Hepatic mRNA expression of the common inflammatory markers *Il6* and *Crp* showed no differences between the two groups, supporting this observation (Figure S1c). Immunohistochemistry (IHC) staining of kidneys revealed a perivascular accumulation of F4/80^+^ macrophages already after 1 and 2 months of HPD, while later on, they mainly accumulated in the periphery of the inflammatory cell clusters (Figure 1c). Significant differences in the F4/80^+^ area between HPD and Ctrl groups emerged already at 2 months and remained evident through 6 months (Figure 1c). After 1 and 2 months of HPD, a few isolated CD45R^+^ B lymphocytes and CD3^+^ T lymphocytes were detected, which appeared in increasing numbers and with increasing cluster accumulation with longer HPD duration (Figure 1d). For CD45R^+^ cells per hpf, differences between HPD and Ctrl groups became apparent after 2 months and reached statistical significance by 4 months. CD3^+^ cell counts showed significant differences between groups as early as 3 months (Figure 1d). This observation was verified by flow cytometry analysis showing a significant increase of lymphocytes from 3 months onwards in HPD mice compared to Ctrl (Figure 1e).

**TABLE 1:** Time dependent biochemical and kidney function parameters, and quantification of kidney injury in C57BL/6N mice on Ctrl and HPD.

Parameter	1 month		2 months		3 months		4 months		6 months	
	Ctrl	HPD	Ctrl	HPD	Ctrl	HPD	Ctrl	HPD	Ctrl	HPD
Serum Pi [mg/dl]	10.8 ± 2.3	14.0 ± 2.5**	10.8 ± 1.9	10.9 ± 1.0	9.7 ± 1.8	14.2 ± 3.2**	9.6 ± 2.4	13.3 ± 4.8*	6.8 ± 1.8	9.0 ± 1.4*
Urinary Pi/crea [mg/mg]	9.6 ± 2.7	36.5 ± 10.1****	4.8 ± 2.6	48.6 ± 5.4****	6.1 ± 2.3	34.3 ± 10.1****	5.5 ± 2.4	30.0 ± 5.3****	7.9 ± 1.7	41.6 ± 6.3****
iFgf23 [pg/ml]	439 ± 73	2541 ± 722****	491 ± 43	4422 ± 827****	158 ± 32	1263 ± 357****	570 ± 61	4298 ± 214****	243 ± 40	2067 ± 624***
Serum crea [mg/dl]	0.18 ± 0.05	0.21 ± 0.08	0.19 ± 0.05	0.24 ± 0.04*	0.18 ± 0.04	0.24 ± 0.03**	0.20 ± 0.05	0.28 ± 0.04**	0.24 ± 0.07	0.34 ± 0.03**
Urinary ACR [mg/g]	13.4 ± 4.3	33.2 ± 14.9***	20.3 ± 14.7	55.0 ± 38.1*	11.6 ± 4.6	29.3 ± 12.4****	17.0 ± 4.8	28.1 ± 11.6**	14.5 ± 6.1	28.7 ± 10.0**
Tubular injury score [a.u.]	0.0 ± 0.0	0.2 ± 0.4	0.0 ± 0.0	1.0 ± 1.0	0.0 ± 0.0	1.3 ± 1.2*	0.0 ± 0.0	1.2 ± 1.3	0.0 ± 0.0	7.3 ± 2.0****
*Havcr1* mRNA [2^−ΔΔCt^]	1.0 ± 0.2	5.4 ± 2.6**	1.0 ± 0.3	6.0 ± 3.9*	1.0 ± 0.3	18.0 ± 14.4**	1.0 ± 0.4	9.8 ± 5.6**	1.0 ± 0.8	46.1 ± 26.4****
Interstitial fibrosis [%]	2.2 ± 0.6	1.9 ± 0.3	1.8 ± 0.3	1.9 ± 0.4	1.2 ± 0.2	1.6 ± 0.5	2.1 ± 1.2	3.3 ± 0.6	4.3 ± 1.1	9.0 ± 1.8***

*Note:* Data are presented as mean ± SD, *n* = 5–10 per group. Data in part from mice in a previously published study [30].

Abbreviations: ACR, albumin to creatinine ratio; crea, creatinine; Ctrl, control phosphate diet; HPD, high phosphate diet; iFGF23, intact fibroblast growth factor 23; Pi, phosphate.

*p < 0.05, **p < 0.01, ***p < 0.001, ****p < 0.0001 compared to respective Ctrl.

### 3.2 HPD Causes an Arterial Dedifferentiation Towards a More Venous Signature, Thereby Promoting the Recruitment of Lymphocytes Through HEV

**FIGURE 2:**
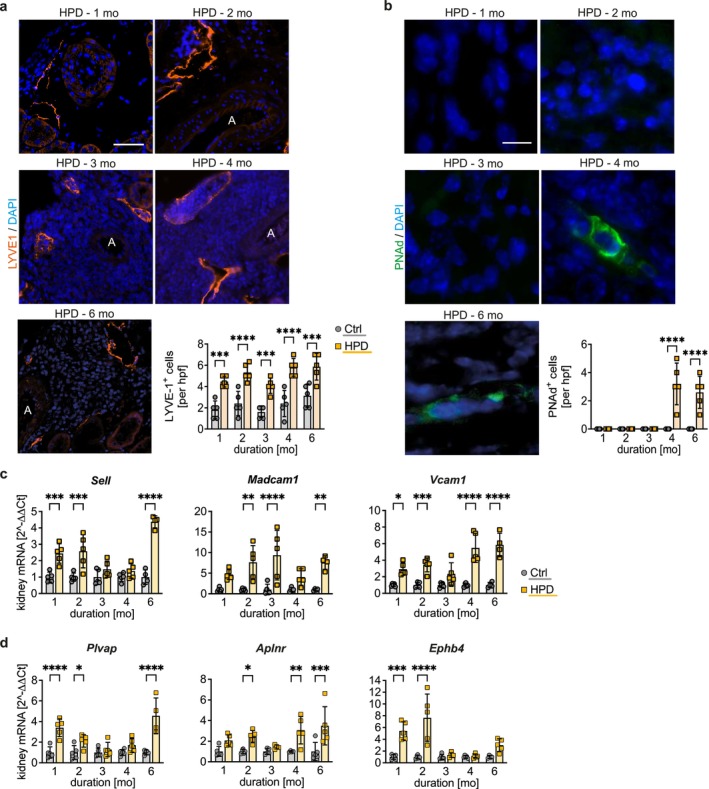
High phosphate diet induces arterial dedifferentiation and lymphatic remodeling, and stimulates the transcription of cell adhesion molecules and venous markers in perivascular TLS. (a) Time‐dependent representative immunofluorescence (IF) staining of LYVE1 (orange) in kidney cross‐sections of mice on high phosphate diet (HPD) Scale bar: 50 μm. The number of LYVE1^+^ cells in HPD and Ctrl group was quantified per high‐power field (hpf). (b) Representative IF staining for PNAd (green) in kidney cross‐sections of mice receiving HPD for 1 up to 6 months. Scale bar: 20 μm. Counterstaining of cell nuclei using DAPI (blue). Quantification of PNAd in HPD and Ctrl group was performed per hpf. (c) Quantitative real‐time PCR analysis for cell adhesion molecules *Sell, Madam1* and *Vcam1*, and (d) venous markers *Plvap, Aplnr* and *Ephb4* in kidney tissue of mice receiving HPD and Ctrl diet. (a–d) Data are presented as the mean ± SD. One‐way analysis of variance (ANOVA) with Sidak's multiple comparisons test, *p < 0.05; **p < 0.01; ***p < 0.001; ****p < 0.0001. A, artery.

We next investigated the vasculature in the regulation of TLS development. Representative images showed that after 2 months of HPD, TLS forming around central arteries were interspersed with LYVE1^+^ lymphatic vessels (Figure 2a). These vessels were partially filled with lymphocytes, an observation not seen in the Ctrl group. Moreover, the number of LYVE1^+^ lymphatic vessels was significantly different between HPD and Ctrl groups as early as 1 month (Figure 2a). At 4 months of HPD onwards, a significantly higher number of PNAd^+^ HEVs was observed (Figure 2b), which is required for lymphocyte leukodiapedesis via L‐selectin (SELL). Whole kidney transcription analyses revealed a marked upregulation of *Sell* and mucosal vascular addressin cell adhesion molecule 1 (*Madcam1*), both important markers for HEV, and of vascular cell adhesion molecule 1 (*Vcam1*), further important for lymphocyte recruitment, in HPD kidneys from 1 month onwards, suggesting an acquisition of high endothelial cell (HEC) function (Figure 2c). This was confirmed by the upregulation of plasmalemma vesicle‐associated protein *Plvap*, apelin receptor *Aplnr*, and ephrin type‐B receptor 4 *Ephb4* already starting at 1 month of HPD (Figure 2d). Taken together, these data suggest a possible shift to the HEC phenotype, indicated by the significantly enhanced expression of markers characterizing arterial dedifferentiation. Thus, the shift of arterial EC identity towards a more venous signature might promote the recruitment of lymphocytes through HEV and finally the formation of TLS.

### 3.3 HPD Stimulates T Cell‐B Cell Interaction and B Cell Transdifferentiation Supporting TLS Maturation

**FIGURE 3:**
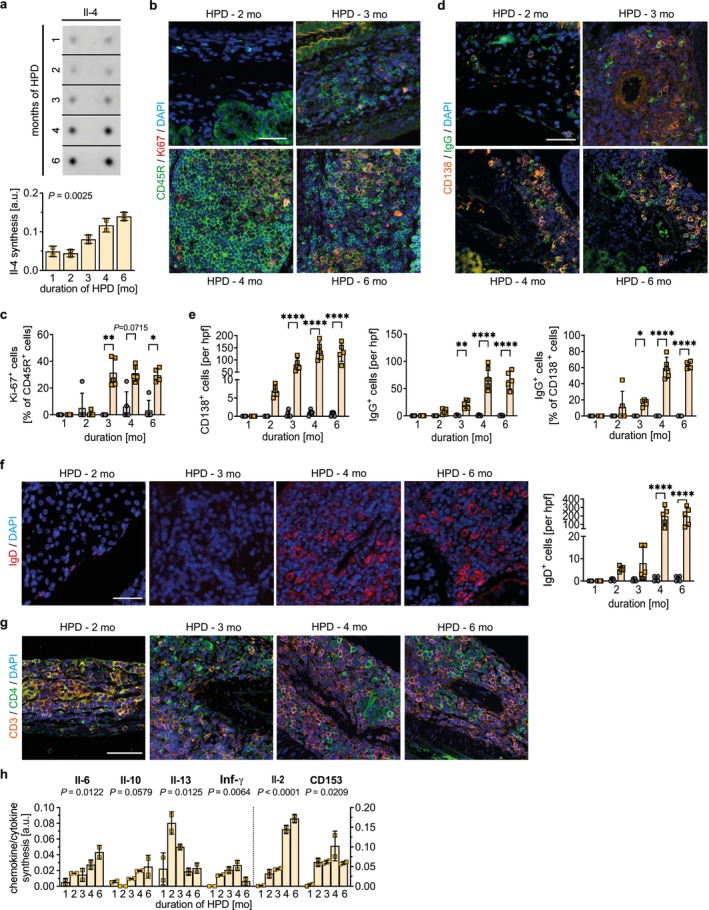
High phosphate loading promotes the interaction of lymphocytes and induces B cell transdifferentiation to advance the maturation of tertiary lymphoid structures (TLS). (a) Cytokine array analysis performed in duplicates of kidney tissue lysates from mice receiving a high phosphate diet (HPD) for 1 up to 6 months showing a progressive increase of interleukin 4 (Il‐4) synthesis. Quantification of Il‐4 spot intensity. Representative immunofluorescence (IF) costaining of (b) CD45R^+^ B cells (green) and the proliferation marker Ki67 (red), (d) for the plasma cell marker CD138 (orange) and IgG (green), (f) for IgD (red), and (g) for T cells positive for CD3 (orange) and CD4 (green), respectively, in kidney tissue cross‐sections of mice on HPD for 2 up to 6 months. (b, d, f) Counterstaining of cell nuclei using DAPI (blue). Scale bars: 50 μm. (c) Ki‐67^+^ cells as a percentage of CD45R^+^ cells were quantified in HPD and Ctrl groups. (e) The number of CD138^+^ cells and IgG^+^ cells in HPD and Ctrl group was quantified per hpf, respectively. In addition, IgG^+^ cells as a percentage of CD138^+^ cells were quantified. (h) Quantification of Il‐6, Il‐10, Il‐13, interferon gamma (Inf‐γ), Il‐2, and CD153 synthesis in kidney tissue lysates from mice receiving a HPD for 1 up to 6 months using spot intensity of cytokine array analyses. (a, c, e, f, h) Data are presented as mean ± SD. One‐way analysis of variance (ANOVA) with Sidak's multiple comparisons test, *p < 0.05; **p < 0.01; ****p < 0.0001.

To further characterize renal TLS development and maturation induced by HPD, cytokine array analysis and immunofluorescence (IF) staining for typical markers of lymphoid tissue cells were performed in kidney cross‐sections. Interleukin 4 (IL‐4), important for lymphocyte proliferation and differentiation [34, 35], was elevated with increasing duration of HPD (Figure 3a). An increasing amount of Ki67^+^ B lymphocytes at 3 months of HPD onwards indicated an active proliferating B cell phenotype supporting the maturation of TLS (Figure 3b,c). Further IF staining for CD138 and IgG observed scattered aggregates of differentiated B lymphocytes into antibody‐producing CD138^+^ plasma cells, the most matured stage of B cell differentiation, starting at 3 months of HPD with increasing cell clustering located around the artery both at 4 and 6 months of HPD. Histological quantification revealed significant differences between HPD and Ctrl in CD138^+^ plasma cells, IgG‐producing cells, and the proportion of IgG^+^ cells among CD138^+^ plasma cells (Figure 3d,e). IF staining and quantification of IgD^+^ B cells (Figure 3f), alongside IgG‐producing plasma cells, proliferating B cells, and follicular dendritic cells, suggest the formation of germinal center‐like structures within the TLS after 4 months of HPD, supporting ongoing B cell activation and maturation. Concomitantly, CD3^+^CD4^+^ T cells predominantly appeared in the first 2 months of HPD, while an increased number of CD3^−^CD4^+^ cells were detectable from the third month onward (Figure 3g). In addition, HPD stimulated the synthesis of interleukins typical for Th2‐CD4^+^ T cells and cytokines, which may point to a possible role for interactions between T cells and B cells (Figure 3h). Taken together, starting with small aggregates of mainly T cells and B cells at perivascular sites in the kidney of HPD mice, the activation of lymphocytes and TLS maturation later on, may have been initiated by antigen presentation and possible T cell‐B cell interactions.

### 3.4 HPD Promotes the Accumulation of Fibroblasts Within TLS Accompanied by Cytokine Production Promoting Lymphocyte Activation and Differentiation

**FIGURE 4:**
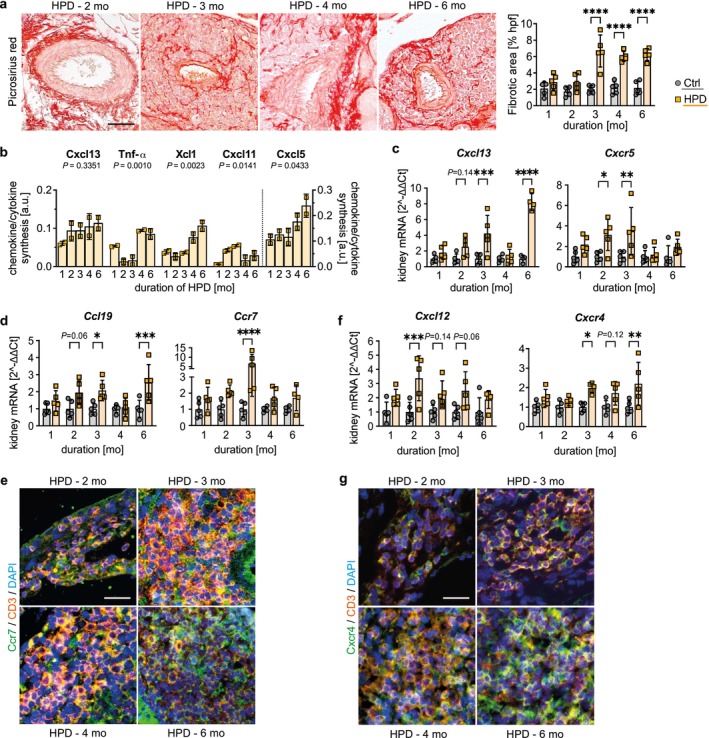
High phosphate load promotes fibroblast accumulation within tertiary lymphoid structure (TLS) accompanied by cytokine production fostering lymphocyte activation and differentiation. (a) Representative picrosirius red‐stained sections from mice receiving a high phosphate diet (HPD) for 1 up to 6 months. Quantification of fibrotic area in percent per hpf. (b) Time‐dependent quantification of Cxcl13, tumor necrosis factor alpha (Tnfα), Xcl1, Cxcl11, and Cxcl5 synthesis in kidney tissue lysates from mice fed with HPD for the indicated time point using spot intensity of cytokine array analyses. Data are presented as mean ± SD. One‐way analysis of variance (ANOVA) with *p* values as indicated. (c) Quantitative real‐time (RT) PCR analysis for (c) *Cxcl13* and its receptor *Cxcr5*, and (d) *Ccl19* and its receptor *Ccr7* in kidney tissue of mice receiving HPD and control diet (Ctrl). (e) Representative immunofluorescence (IF) costaining for Ccr7 (green) and CD3 (orange) in kidney tissue cross‐sections of mice on HPD for 2 up to 4 months. (f) Quantitative RT‐PCR analysis for *Cxcl12* and its receptor *Cxcr4* in mice on Ctrl and HPD. (g) Representative IF costaining for Cxcr4 (green) and CD3 (orange) in kidney tissue cross‐sections of mice on HPD for 2 up to 4 months. (e, g) Counterstaining of cell nuclei using DAPI (blue). Scale bars: 50 μm. (a–f) Data are presented as mean ± SD. One‐way analysis of variance (ANOVA) with Sidak's multiple comparisons test, *p < 0.05; **p < 0.01; ***p < 0.001; ****p < 0.0001.

We next investigated the transdifferentiation of perivascular resident fibroblasts into chemokine‐producing immunofibroblasts within the TLS, known to stimulate the recruitment of immune cells [36, 37]. In areas of lymphocyte infiltration around a central artery, picrosirius red staining showed a progressive accumulation of local interstitial matrix deposition and of fibrotic fibers. Significant differences in fibrotic area were observed between HPD and Ctrl groups starting at 3 months (Figure 4a). Array analysis of HPD kidney lysates observed time‐dependent induction of chemokines and cytokines (Figure 4b) known to be involved in fibroblast phenotypic shift or expressed by transdifferentiated fibroblasts to support lymphocyte accumulation. Although ANOVA analysis showed no statistically significant trend on protein level, the homeostatic C‐X‐C motif ligand (Cxcl) 13, produced by immunofibroblasts and mainly attracting B lymphocytes [2, 16], was enhanced with increasing duration of HPD and significantly upregulated after 3 months on transcriptional level (Figure 4c). Concomitantly, the expression of the Cxcl13 receptor *Cxcr5* followed the same upregulation (Figure 4c). The transcription of the C‐C motif ligand 19 (*Ccl19*), also synthesized by immunofibroblasts and mainly triggering T lymphocyte recruitment [38, 39], and its receptor *Ccr7* were markedly upregulated with increasing duration of HPD (Figure 4d). IF double staining verified an increasing number of Ccr7^+^CD3^+^ T cells (Figure 4e). The molecular structure of HPD‐mediated TLS development was corroborated by the increasing transcription of *Cxcl12* (Figure 4f), which is involved in the recruitment of Cxcr4^+^ lymphocytes [21, 40]. This observation was confirmed by the increased accumulation of Cxcr4^+^CD3^+^ lymphocytes (Figure 4g). In conclusion, due to a high phosphate load, fibroblasts accumulated within TLS and the detected homeostatic chemokines and cytokines, known to promote lymphocyte activation and differentiation, might in turn facilitate a phenotypic shift of perivascular fibroblasts into immunofibroblasts.

### 3.5 Development of FDC and FRC Networks in HPD‐Induced TLS

**FIGURE 5:**
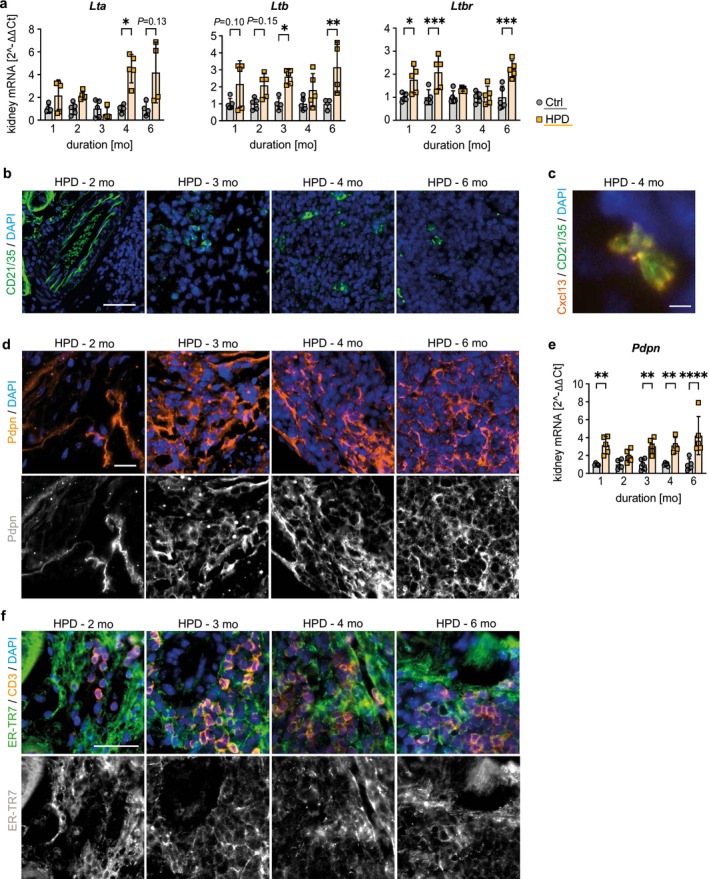
Development of FDC and FRC Networks in HPD‐induced TLS. (a) Quantitative RT‐PCR analysis for lymphotoxins *Lta* and *Ltb* and its receptor *Ltbr* in mice on Ctrl and HPD. Representative IF costaining of (b) CD21/35^+^ follicular dendritic cells (FDCs; green), (c) for Cxcl13 (orange) and CD21/35 (green), and (d) for podoplanin^+^ cells (Pdpn; orange) in kidney tissue cross‐sections of mice on HPD for 2 up to 4 months. (e) Quantitative RT‐PCR analysis for *Pdpn* in mice on Ctrl and HPD. (f) Representative IF costaining of ER‐TR7^+^ follicular reticular cells (FRCs, green) and CD3 (orange) in kidney tissue cross‐sections of mice on HPD for 2 up to 4 months. (b–d, f) Counterstaining of cell nuclei using DAPI (blue). Scale bars: 50 μm, except for (c) 2 μm. (a, e) Data are presented as mean ± SD. One‐way analysis of variance (ANOVA) with Sidak's multiple comparisons test, *p < 0.05; **p < 0.01; ***p < 0.001; ****p < 0.0001.

The mRNA expression of lymphotoxin (Lt) alpha (*Lta*) and Lt beta (*Ltb*), secreted by both B and T cells, and Ltb receptor (*Ltbr*) was upregulated in kidney tissue of HPD mice at almost all time points except *Ltbr*, which was significantly induced at the beginning only (Figure 5a). During TLS maturation, lymphotoxins and tumor necrosis factor alpha (TNF‐α) are known to promote the transdifferentiation of immunofibroblasts into antigen‐representing FDC thereby stabilizing B cell clustering [41, 42]. In parallel to the increasing expression of lymphotoxins and synthesis of Tnf‐α (Figure 4b) with extended duration of HPD, a higher number of CD21/35^+^ FDCs was also observed (Figure 5b), which were also positive for Cxcl13 (Figure 5c), further supporting B cell activation and transdifferentiation into germinal center B cells. While factors initiating the differentiation are still not known, immunofibroblasts can further transdifferentiate into FRCs, which form a network to transport antigens and signaling molecules and support the migration of T lymphocytes [23, 43, 44]. Already by 2 months of HPD, a network of podoplanin‐expressing (Pdpn^+^) FRCs was observed throughout the entire TLS (Figure 5d). The *Pdpn* mRNA expression was already significantly enhanced at 1 month of HPD compared to Ctrl underlining the importance of FRCs from the very beginning of TLS formation (Figure 5e). In addition, stroma cells that stained positive for the FRC marker ER‐TR7 showed a conduit network organized in CD3^+^ lymphocyte areas of the TLS starting at 2 months of HPD (Figure 5f). Taken together, the increased mRNA expression of *Lta*, *Ltb* and *LtbR* might promote FDC differentiation and B cell cluster formation in the HPD group with stromal networks expressing podoplanin and ER‐TR7.

### 3.6 High Phosphate Rather Than High FGF23 Promotes Tubular Injury and the Formation of Perivascular TLS

**FIGURE 6:**
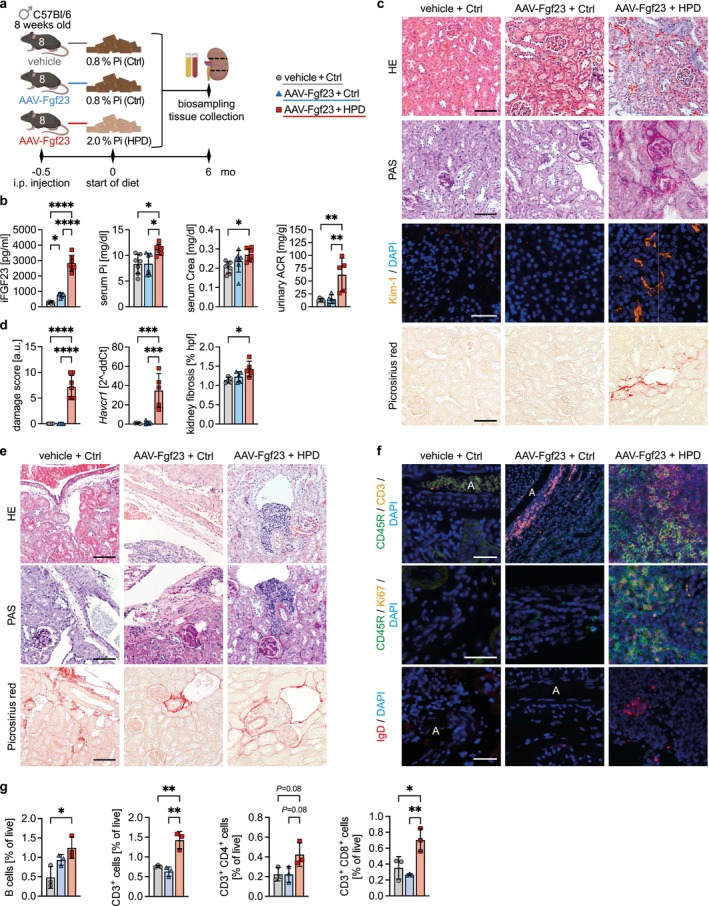
High phosphate, not the increase of fibroblast growth factor 23 (FGF23), drives tubular injury and perivascular tertiary lymphoid structure (TLS) formation. (a) Schematic representation of the study design including specific time points for adeno‐associated virus (AAV) treatment inducing the overexpression of fibroblast growth factor 23 (FGF23), dietary intervention, as well as biosampling and tissue collection. (b) Quantification of plasma intact FGF23 (iFGF23) concentrations, serum phosphate (Pi), serum creatinine (Crea), and urinary albumin to creatinine ratio (ACR) in all three groups. (c) Representative images of the cortex of hematoxylin and eosin (HE), periodic acid‐Schiff (PAS), and picrosirius red stained kidney cross‐sections, as well as immunofluorescence (IF) staining of kidney injury molecule 1 (Kim‐1; orange) positive tubules. (d) Quantification of the scoring of tubular injury, of real‐time PCR analysis of *Havcr1*, and of kidney fibrosis in the cortex. (e) Representative HE, PAS and picrosirius red staining focusing on the perivascular region of TLS development. (f) Representative IF costaining of CD45R^+^ (green) and CD3^+^ (orange) lymphocytes, of CD45R (green) and Ki67 (orange), and of IgD (red) synthesis. (g) Quantification of lymphocytes using flow cytometry analysis of whole kidney tissue from mice of all three groups. (c, f) Counterstaining of cell nuclei using DAPI (blue). (c, e and f) Scale bars: 100 μm. (b, d, g) Data are presented as mean ± SD. One‐way analysis of variance (ANOVA) with Tukey's multiple comparisons test, *p < 0.05; **p < 0.01; ***p < 0.001; ****p < 0.0001. A, artery.

We next investigated if tubular injury and/or TLS development was caused directly by high phosphate and/or by the increased plasma FGF23 levels. To this aim, we induced high circulating Fgf23 concentrations in mice by an adeno‐associated virus (AAV)‐mediated approach as described previously [31], fed these mice with either HPD or Ctrl diet for 6 months and compared the kidney phenotypes with vehicle‐treated mice on Ctrl diet (Figure 6a). As expected, plasma FGF23 levels were significantly higher in AAV‐Fgf23 mice compared to vehicle‐treated mice (AAV‐ctrl) on Ctrl diet and further increased in AAV‐Fgf23 mice by concomitant HPD treatment, while serum phosphate was elevated in AAV‐Fgf23 + HPD mice only (Figure 6b). Interestingly, serum Crea and urinary ACR were enhanced in AAV‐Fgf23 + HPD mice only, suggesting that high phosphate caused impaired kidney function rather than FGF23. On a cellular level, scoring of HE and periodic acid Schiff (PAS)‐stained kidney sections revealed an induction of proximal tubular damage in AAV‐Fgf23 + HPD mice only that was characterized by the loss of epithelial cell polarity and impaired brush border membrane integrity (Figure 6c,d). Concomitantly, staining for the tubule‐specific kidney injury marker 1 (Kim‐1) showed strong positive signals localized to areas of damaged tubules in the kidney cortex of HPD‐fed AAV‐Fgf23 mice, which were confirmed by significantly upregulated *Havcr1* transcription (Figure 6c,d). Tubular damage was accompanied by an increased accumulation of collagen fibers detected by picrosirius red staining and quantification observed enhanced interstitial fibrosis in the AAV‐Fgf23 group on HPD only (Figure 6c,d).

With the focus on renal TLS formation and development, HE and PAS staining showed an accumulation of mononuclear cells in AAV‐Fgf23 on Ctrl diet compared to vehicle‐treated mice, but this was not associated with a central artery (Figure 6e). In contrast, concomitant feeding of AAV‐Fgf23 mice with HPD caused the formation of perivascular TLS, as previously observed. Further, picrosirius red staining indicated the formation of collagen networks within the TLS (Figure 6e). IF staining revealed that immune cell aggregates in the kidneys of AAV‐Fgf23 + Ctrl mice consisted of CD3^+^ T lymphocytes, but not of CD45R^+^ B lymphocytes, while HPD caused the accumulation of both CD45R^+^ B cell and CD3^+^ T cell clusters, respectively (Figure 6f). The detection of proliferating Ki‐67^+^ B cells and the synthesis of IgD confirmed the maturation of TLS in the kidneys of AAV‐Fgf23 + HPD mice only (Figure 6f). Quantified by flow cytometry, the amount of T and B lymphocytes was higher in AAV‐Fgf23 + HPD mice compared to both groups on Ctrl diet (Figure 6g). Taken together, our data suggest that a chronic high phosphate load directly, and not via increased FGF23 concentration, causes proximal tubular damage with tubulointerstitial fibrosis and the concomitant formation of perivascular TLS.

### 3.7 High FGF23 in the Setting of Hypophosphatemia Does Not Induce Kidney Damage or TLS Development

**FIGURE 7:**
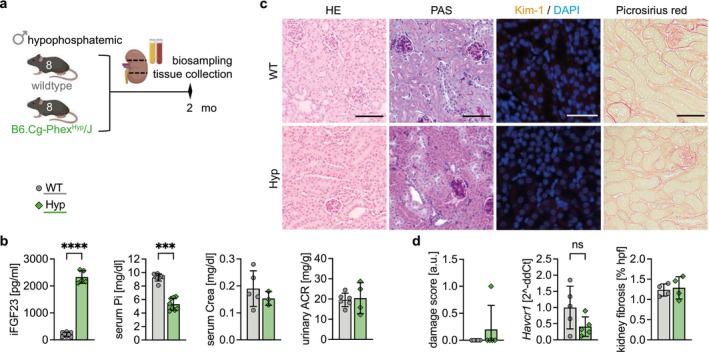
Elevated fibroblast growth factor 23 (FGF23) levels in hypophosphatemic mice do not cause kidney damage or promote the formation of tertiary lymphoid structures (TLS). (a) Schematic representation of the study design including time point for biosampling and tissue collection. (b) Quantification of plasma intact FGF23 (iFGF23) concentrations, serum phosphate (Pi), serum creatinine (Crea), and urinary albumin to creatinine ratio (ACR) in all three groups. (c) Representative images of the cortex of hematoxylin and eosin (HE), periodic acid‐Schiff (PAS), and picrosirius red stained kidney cross‐sections, as well as immunofluorescence (IF) staining of kidney injury molecule 1 (Kim‐1; orange) with DAPI (blue) counterstaining. Scale bars: 100 μm. (d) Quantification of the scoring of tubular injury, of real‐time PCR analysis of *Havcr1* transcription, and of kidney fibrosis in the cortex. (b, d) Data are presented as mean ± SD. Unpaired *t*‐tests with ****p < 0.0001.

We next investigated the kidney phenotype in 2‐month‐old hypophosphatemic B6.Cg‐*Phex* ^*Hyp*^/J (*Hyp*) mice, an animal model of X‐linked hypophosphatemia (Figure 7a). As expected, *Hyp* mice showed significantly elevated FGF23 levels and a concomitant hypophosphatemia compared to wildtype (WT) littermates (Figure 7b). Neither serum Crea nor urinary ACR was significantly different between *Hyp* and WT mice, indicating normal kidney function (Figure 7b). HE and PAS staining of kidney cross‐sections revealed no damage to proximal tubules and the tubular damage scores did not statistically differ between groups (Figure 7c,d). Kim‐1^+^ tubules were neither detected in the kidneys of WT nor of *Hyp* mice and this observation aligned with no alterations in *Havcr1* transcription (Figure 7c,d). Picrosirius red staining and quantification of collagen fibers showed no signs of interstitial fibrosis in both groups (Figure 7c,d) and no immune cell accumulation could be detected. Taken together, high FGF23 levels in the setting of hypophosphatemia do neither induce tubular injury nor promote the accumulation of immune cells or the formation of TLS.

## 4 Discussion

**FIGURE 8:**
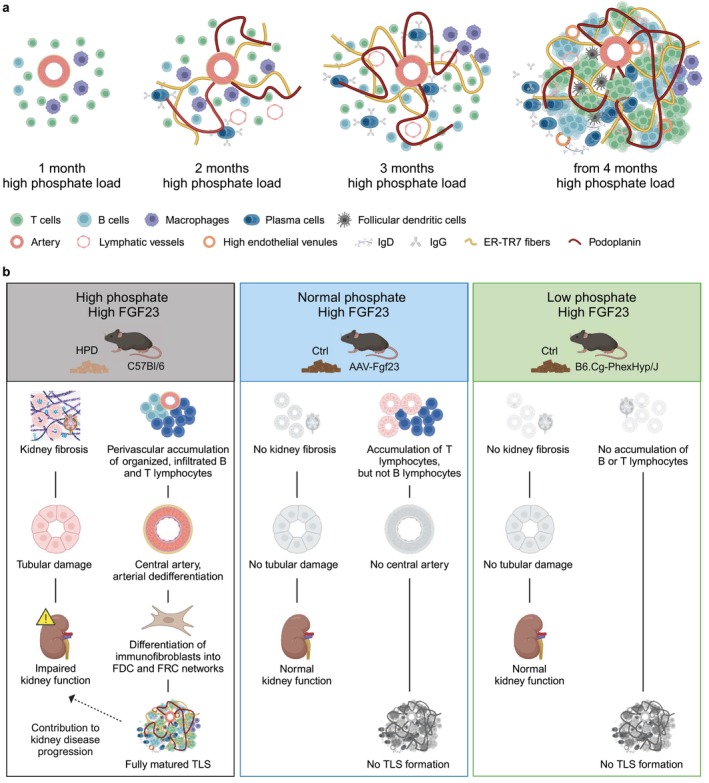
Schematic representation of the development of tertiary lymphoid structures (TLS) and tubular injury due to high phosphate loading. (a) After 1 month of high phosphate diet (HPD), scattered T cells, B cells, and macrophages begin to accumulate around a central artery. By 2 months, larger clusters of B and T cells and individual IgG‐producing plasma cells are found. Additionally, lymphatic vessels are observed, and podoplanin^+^ and ER‐TR7^+^ cells start to form networks within the TLS. After 3 months of HPD, larger clusters of T and B cells are organized within the TLS, macrophages accumulate at the edges, and podoplanin^+^ and ER‐TR7^+^ cells form honeycomb‐like networks through the TLS. By 4 months, TLS is fully matured and shows distinct T and B cell areas with T cells primarily surrounded by fibroblastic reticular cell (FRC) networks and follicular dendritic cells (FDC) present in B cell clusters. High endothelial venules and lymphatic vessels remain within the TLS, and both IgG and IgD are present. (b) In mice exposed to HPD, phosphate and FGF23 levels increase, and kidney fibrosis, tubular damage, and impaired kidney function are observed with concomitant perivascular accumulation of organized infiltrates of B and T cells, along with the development of FRC and FDC networks, indicating the formation of fully mature TLS (left panel). Overexpression of FGF23 alone increases FGF23 levels, while phosphate remains normal, and no fibrosis, tubular damage, or impaired kidney function is observed. Some lymphocytes accumulate in tubular regions, but without forming TLS (middle panel). *Hyp* mice have low phosphate but high FGF23 levels and show neither kidney fibrosis, tubular damage, nor any signs of kidney impairment, nor accumulation of lymphocytes or TLS formation (right panel). Created with BioRender.

This study gives evidence that a high phosphate directly leads to the progression of kidney damage and a chronic inflammatory environment, thereby promoting the formation of TLS. High phosphate, in parallel with tubular injury and fibrosis, induces perivascular accumulation of organized, infiltrated CD45R^+^ B and CD3^+^ T lymphocytes in the kidney and causes arterial dedifferentiation, thereby promoting the recruitment of lymphocytes through PNAd^+^ HEVs and lymphatic vessels (Figure 8a,b). Our results suggest that high phosphate stimulates the interaction of T cells and B cells leading to B cell transdifferentiation into antibody‐producing plasma cells with germinal center‐like structures and differentiation of perivascular fibroblasts into CD21/35^+^ FDC and Pdpn^+^ FRC networks indicating formation of mature TLS. Our study emphasizes the pathophysiological role of chronically high phosphate loading in progressive kidney disease.

Previous studies indicate that TLS formation usually originates from perivascular areas [18, 45, 46, 47]. Limbourg et al. demonstrated that the absence of Notch signaling in vascular endothelial cells resulted in the spontaneous formation of TLS in mouse kidneys, whereby arteries acquired an HEV‐like phenotype facilitating the recruitment and infiltration of lymphocytes [22]. This was also the case in the present study, where infiltrating lymphocytes accumulated around large arteries in the corticomedullary zone in kidneys of HPD mice beginning as early as 1 month of high phosphate load. This suggests that high phosphate levels cause damage to the arterial endothelium, which in turn led to the release of cytokines increasing the recruitment of lymphocytes. Intriguingly, HPD‐mediated accumulation of inflammatory cell clusters was kidney specific and neither found in other organs such as liver or heart. High phosphate induced lymphangiogenesis in the kidney, renal vasculature acquired characteristics of HEV supporting extravasation of lymphocytes, and enhanced transcription of *Ephb4* and *Aplnr* in whole kidney tissue, all indicating the transition of renal endothelial cells from an arterial to a venous phenotype [48, 49, 50, 51]. We further observed an increased expression of *Plvap*, a vascular marker associated with leukocyte recruitment, in the first months of HPD, supporting an early immune cell aggregation. The importance of Plvap in the context of transcellular leukocyte migration under inflammatory conditions has already been discussed by Keuschnigg et al. demonstrating that blocking Plvap via antibody decreased the migration of leukocytes in a model of peritonitis [52].

As we demonstrated increased transcription of venous markers in whole kidney tissue of HPD mice, we additionally investigated the presence and maturation of HEVs as a strong criterion of TLS formation [17, 36]. The mRNA expression of *Madcam1*, a marker of early immature HEVs [53], progressively increased from 1 up to 3 months of HPD, supporting an early beginning of TLS formation. Consistently, IF staining showed PNAd^+^ HEVs from 4 months HPD onwards, indicating the differentiation of HEVs to an adult mature phenotype [54]. These findings support that the migration of immune cells in the first few months of HPD had to primarily take place via lymphatic vessels in the kidney. Finally, we observed that high phosphate loading increased the presence of Lyve‐1^+^ lymphatic vessels in the TLS as early as the second month suggesting the production of lymphangiogenic growth factors.

CD3^−^CD4^+^ cells in TLS are defined as potential lymphoid tissue inducer (LTi) cells, but their role in TLS organization and function is controversially discussed [55]. Here, we detected an increased number of CD3^−^CD4^+^ cells in kidney TLS in addition to the enhanced synthesis of interleukins (Il‐2, Il‐6, Il‐10, Il‐13) and cytokines (Inf‐γ, CD153) that are discussed to support the interaction of activated T cells with B cells further promoting B cell transdifferentiation into germinal center follicular B cells [55, 56, 57]. Thus, our results indicate the presence of LTi cells in kidney TLS induced by HPD. However, further studies, especially the concise characterization of this cell type are necessary to determine their possible role in T cell‐B cell interaction.

Several studies discuss the importance of pro‐inflammatory cytokines that trigger the differentiation of fibroblasts into immunofibroblasts, and thus favor the aggregation of immune cells and promote the development of TLS [58, 59, 60, 61, 62]. Primed fibroblasts and stromal cells can release Cxcl13 and Ccl19 as well as VCAM1, all of which further support recruitment and organization of inflammatory cells within the TLS [16, 42, 63]. The chemokine Cxcl13 binds to B cells and subsets of T cells through the Cxcr5 receptor and thereby regulates cell homing and structural organization of the TLS [2, 64, 65]. Cxcl13 has been described in the formation of B cell infiltrates in acute renal transplant rejection, interstitial nephritis and IgA nephropathy as well as in an animal model of lupus nephritis [66, 67, 68, 69]. The deletion of its receptor Cxcr5 prevented the de novo formation of ectopic lymphoid follicles in a *helicobacter pylori* mouse model [70], further indicating the importance of the Cxcl13/Cxcr5 interaction for TLS formation. Ccl19 binds to the Ccr7 receptor on B and T cells and is involved in the organization of T cell regions as well as the recruitment of lymphocytes [61, 71, 72, 73]. The cytokine Cxcl12 promotes the recruitment of Cxcr4^+^ T cells [21]. Here we showed by array analysis an increased synthesis of cytokines promoting T cell–B cell interaction and B cell antibody production in kidneys of HPD from 1 month onwards, suggesting a fibroblast phenotypic shift triggering the maturation of TLS. Cxcl5 increased over time of dietary intervention, which has previously been described in the context of fibroblast priming [61]. HPD significantly increased the expression levels of *Cxcl13*, *Ccl19, Cxcl12* and *Vcam1* within the first 3 months, indicating their relevance for the recruitment of lymphocytes in the formation of TLS. Additionally, transcription analysis showed increasing expression of *Cxcr5*, *Ccr7* and *Cxcr4* receptors, concomitantly.

Lymphotoxins are members of the TNF superfamily of cytokines and play a critical role during organogenesis of secondary lymphatic organs [2, 74, 75]. Lymphotoxins and TNFα promote the transdifferentiation of immunofibroblasts into FDCs [41]. Thereby it is also discussed that activation of LTbR signaling can induce Cxcl13, further supporting the differentiation and formation of FDC networks essential for the internal organization and structure of lymphoid organs [58, 76]. However, the role of LTbR signaling in TLS development is controversially discussed [23, 37, 77, 78, 79]. Even though recent studies have shown that TLS can develop independently of LTbR signaling, animal models lacking a functioning LTbR signaling presented with increasingly disorganized TLS over time [42]. In the present study, HPD stimulated the synthesis of TNFα and Cxcl13 and caused an upregulation of lymphotoxins in whole kidney tissue until the fourth month of dietary intervention, while *Ltbr* transcription was significantly induced at the beginning only, suggesting a possible role of LTbR signaling in the formation of TLS.

Depending on their exposition to specific cytokines, chemokines and lymphotoxines, immunofibroblasts undergo a phenotypic shift to FDCs or FRCs, where Pdpn^+^ FRC networks are formed within T cell regions and CD21/35^+^ FDC networks are mainly observed in B cell regions [23, 80]. FRCs are immunologically specialized Pdpn^+^ myofibroblasts and are able to create a cell infrastructure to transport antigens and signaling molecules and support immune cell migration found in lymph nodes, spleen and thymus [81]. Thereby, FRCs form a net of cell–cell contacts and create a conduit network by producing components of the extracellular matrix. Peduto et al. showed that under inflammatory conditions, local fibroblasts transform into Pdpn^+^ immunofibroblasts after exposition to stroma cell‐derived proinflammatory cytokines, such as Cxcl13, Ccl19 and Vcam1 [37], which were also induced early in HPD fed mice in our study. In contrast, the deactivation of Pdpn^+^ fibroblasts significantly restricted the development of TLS by AdV5 infection in salivary glands [42]. Limbourg et al. described the formation of conduit networks of stromal cells and fibroblasts that were positively stained for the FRC markers Pdpn and ER‐TR7 and extended in a honeycomb pattern from a central artery to the periphery [22]. Accordingly, Pdpn^+^ and ER‐TR7^+^ networks formed due to high phosphate exposure, which started 2 months after HPD. ER‐TR7^+^ structures were surrounded by CD3^+^ T cells further confirming the formation of conduit systems, which underlines the involvement of primed immunofibroblasts in the high phosphate model. While FRC networks were already observed at 2 months of HPD, FDC networks appeared in mature TLS after 4 months of HPD.

The role of TLS in organ function in pathology still remains unclear. Several studies indicate that renal TLS need to be considered as a pathological parameter for disease severity or poor renal condition [11, 12, 14, 20, 21]. However, mechanistic insights into the pathological signaling of TLS during (kidney) disease progression are still unclear. Luo et al. have demonstrated that inhibiting the formation of renal TLS after kidney injury in aged mice by blocking lymphocyte accumulation with fingolimod (FTY720) treatment, attenuated intrarenal inflammation and fibrosis, suggesting a pathological role of TLS in kidney injury progression [11]. Sekine et al. reported that plasma cells directly contribute to tissue destruction and loss of organ function in the inflamed kidney due to the secretion of antibodies [82]. In the present study, a high phosphate load caused a continuous progression of tubular damage, fibrosis and TLS development with differentiation of B cells into CD138^+^, IgG and IgD producing plasma cells within the TLS starting at 3 months of HPD. From the fourth month onwards, mature TLS persisted, while tubular damage and fibrosis progressed, with a markedly increased rate between the fifth and sixth months of HPD. Although we cannot determine which occurs first, our data suggest that autoantibody‐producing mature TLS may contribute to further progression of kidney disease by binding of autoantibodies to proximal tubule cells and stimulating cytokine production, leading to a virtuous cycle of inflammation and tubular damage.

Our results suggest that phosphate itself and not the induction of the phosphaturic hormone FGF23 is the main cause of progressive kidney injury and TLS formation in high phosphate loading. In this regard, genetically modified *Fgf23* overexpressing mice only developed mature TLS accompanied by tubular damage and increased fibrosis when receiving HPD. Likewise, in hypophosphatemic *Hyp* mice, no immune cell aggregates nor kidney damage were observed despite elevated FGF23 levels. This is underscored by the observation that stimulation of human proximal tubule epithelial cells with high phosphate upregulates the transcription of the kidney injury marker *Havcr*1, whereas treatment with FGF23 does not [30], further supporting the concept that high phosphate directly promoted the observed kidney pathologies in the present study. Taken together, our study emphasizes the pathophysiological importance of chronically high phosphate in the pathogenesis of progressive kidney disease and TLS formation.

### 4.1 Limitations of the Study

The present study has some limitations. Even by limiting the use of kidney tissue to only the middle disk, it might be possible that at some point the analyzed tissue parts did not contain TLS. Furthermore, the observations in this study are not functionally proven. Necessarily, our hypothesis needs to be addressed in further studies using adequate models and methods to identify the mechanism behind. Moreover, to better understand the changes happening in renal tissue during high phosphate load, RNA‐sequencing studies should be performed.

## Author Contributions

M.L.‐N. performed conceptualization. N.W. and F.W. established the methodology and carried out investigations. N.W. and B.R. performed the evaluations and statistical analyses. T.K. performed flow cytometry analysis. J.S. and J.H.B. scored tubular injury. F.P.L. and D.H. critically discussed the study design and data. N.W. and B.R. wrote the original draft of the manuscript. B.R., N.W. and M.L.‐N. wrote, reviewed and edited the manuscript. D.H. intensively reviewed and edited the manuscript. N.W., B.R., and M.L.‐N. performed visualization. B.R. and M.L.‐N. conducted supervision. F.P.L. provided resources. D.H. provided resources and performed funding. All authors read, edited and approved the manuscript.

## Funding

The authors have nothing to report.

## Conflicts of Interest

The authors declare no conflicts of interest.

## Supporting information


**Figure S1:** (a) Representative HE stained sections of liver tissue from the control (Ctrl) and HPD group after 6 months with no detectable formation of TLS. Scale bar: 100 um. (b) Representative HE stained sections of heart tissue from both Ctrl and HPD groups, showing no TLS formation in either group. Scale bar: 100 um. (c) Quantitative real‐time PCR analysis of C‐reactive protein (Crp) and interleukin 6 (Il6) in both groups after six months dietary intervention. Data are presented as the mean+/‐ SD. Unpaired t‐tests with *p* > 0.05. (d) Representative immunofluorescence co‐staining of CD45R+ B cells (green) and CD3+ T cells (orange) showing the accumulation and distinct separation of B and T cell areas during the different stages of TLS developement in kidney tissue cross‐sections of mice on HPD for one up to six months. Counterstaining of cell nuclei using DAPI (blue). Scale bar: 50 um.

**Table S1:** Primary antibodies for histology staining.
**Table S2:** Secondary antibodies for histology staining.
**Table S3:** Primer sequences for qRT‐PCR.

## Data Availability

The data that support the findings of this study are available in the Results and Supporting Information of this article.
