# Supplementary figures and images for: High Phosphate Load Induces De Novo Formation of Tertiary Lymphoid Structures in the Kidney
Source: FASEB J. 2025 Dec 12;39(24):e71279. doi: 10.1096/fj.202500968R (PMC12700132; doi:10.1096/fj.202500968R)

# Supplemental Figure 1

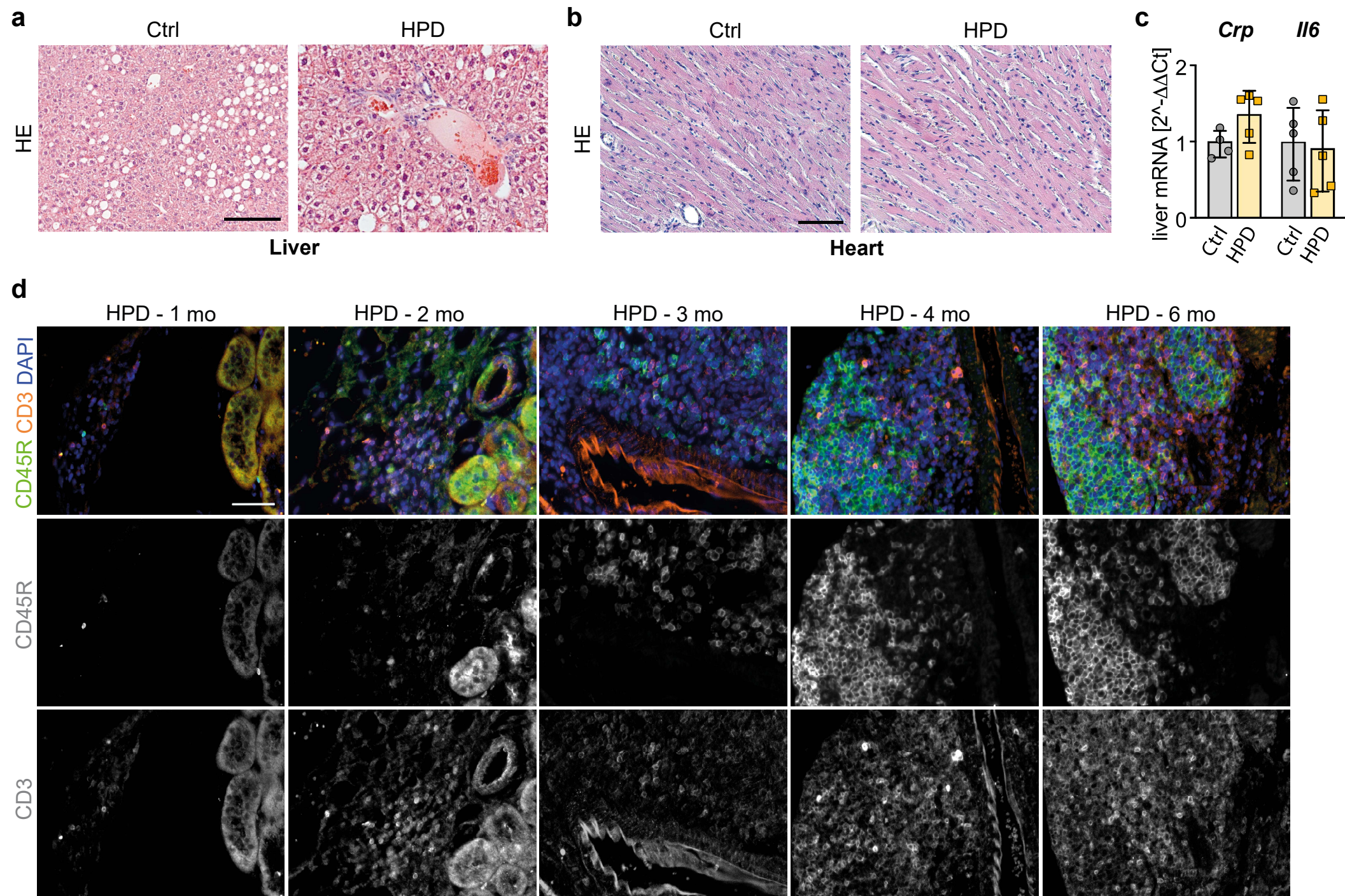

Supplement: Supplementary file 1 — Figure S1: (a) Representative HE stained sections of liver tissue from the control (Ctrl) and HPD group after 6 months with no detectable formation of TLS. Scale bar: 100 um. (b) Representative HE stained sections of heart tissue from both Ctrl and HPD groups, showing no TLS formation in either group. Scale bar: 100 um. (c) Quantitative real‐time PCR analysis of C‐reactive protein (Crp) and interleukin 6 (Il6) in both groups after six months dietary intervention. Data are presented as the mean+/‐ SD. Unpaired t‐tests with p > 0.05. (d) Representative immunofluorescence co‐staining of CD45R+ B cells (green) and CD3+ T cells (orange) showing the accumulation and distinct separation of B and T cell areas during the different stages of TLS developement in kidney tissue cross‐sections of mice on HPD for one up to six months. Counterstaining of cell nuclei using DAPI (blue). Scale bar: 50 um. [file FSB2-39-e71279-s001.pdf]
