# Supplementary material for: High Phosphate Load Induces De Novo Formation of Tertiary Lymphoid Structures in the Kidney
Source: FASEB J. 2025 Dec 12;39(24):e71279. doi: 10.1096/fj.202500968R (PMC12700132; doi:10.1096/fj.202500968R)
Supplement: Supplementary file 2 — Table S1: Primary antibodies for histology staining. Table S2: Secondary antibodies for histology staining. Table S3: Primer sequences for qRT‐PCR. [file FSB2-39-e71279-s002.docx]

# Supplementary Information

**High phosphate load induces de novo formation of tertiary lymphoid structures in the kidney**

Nina Weingärtner^1§^, Beatrice Richter, PhD^1§^, Franziska Walles^1^, Tamar Kapanadze, PhD^2,3^, Jessica Schmitz^4^, Jan H. Bräsen, MD^4^, Florian P. Limbourg, MD^2,3^, Dieter Haffner, MD^1^, Maren Leifheit-Nestler, PhD^1^*

^1^Department of Pediatric Kidney, Liver, Metabolic and Neurological Diseases, Pediatric Research Center, Hannover Medical School, Hannover, Germany.

^2^Department of Nephrology and Hypertension, Hannover Medical School, Hannover, Germany.

^3^Vascular Medicine Research, Hannover Medical School, Hannover, Germany.

^4^Institute of Pathology, Nephropathology Unit, Hannover Medical School, Hannover, Germany.

^§^Authors contributed equally to this work

* Lead contact.

**Correspondence:** leifheit-nestler.maren@mh-hannover.de

**Supplementary Figure**

**Supplementary Figure S1**

**Supplemental Figure 1:** (**a**) Representative HE stained sections of liver tissue from the control (Ctrl) and HPD group after six months with no detectable formation of TLS. Scale bar: 100 µm. (**b**) Representative HE stained sections of heart tissue from both Ctrl and HPD groups, showing no TLS formation in either group. Scale bar: 100 µm. (**c**) Quantitative real-time PCR analysis of C-reactive protein (Crp) and interleukin 6 (Il6) in both groups after six months dietary intervention. Data are presented as the mean ± SD. Unpaired t-tests with P > 0.05. (**d**) Representative immunofluorescence co-staining of CD45R^+^ B cells (green) and CD3^+^ T cells (orange) showing the accumulation and distinct separation of B and T cell areas during the different stages of TLS development in kidney tissue cross-sections of mice on HPD for one up to six months. Counterstaining of cell nuclei using DAPI (blue). Scale bar: 50 µm.
